# Probiotic lactic acid bacteria promote anti-tumor immunity through enhanced major histocompatibility complex class I-restricted antigen presentation machinery in dendritic cells

**DOI:** 10.3389/fimmu.2024.1335975

**Published:** 2024-03-27

**Authors:** Suguru Saito, Alato Okuno, Zhenzi Peng, Duo-Yao Cao, Noriko M. Tsuji

**Affiliations:** ^1^ Division of Cellular and Molecular Engineering, Department of Life Technology and Science, National Institute of Advanced Industrial Science and Technology (AIST), Tsukuba, Ibaraki, Japan; ^2^ Division of Virology, Department of Infection and Immunity, Faculty of Medicine, Jichi Medical University, Shimotsuke, Tochigi, Japan; ^3^ Department of Biomedical Sciences, Cedars-Sinai Medical Center, Los Angeles, CA, United States; ^4^ Department of Health and Nutrition, Faculty of Human Design, Shibata Gakuen University, Hirosaki, Aomori, Japan; ^5^ Department of Cell Biology and Genetics, School of Basic Medical Sciences, Hengyang Medical School, University of South China, Hengyang, Hunan, China; ^6^ Division of Immune Homeostasis, Department of Pathology and Microbiology, Nihon University School of Medicine, Tokyo, Japan; ^7^ Division of Microbiology, Department of Pathology and Microbiology, Nihon University School of Medicine, Tokyo, Japan; ^8^ Department of Food Science, Jumonji University, Niiza, Saitama, Japan

**Keywords:** probiotics, lactic acid bacteria, dendritic cells, CD8+ T cells, anti-tumor immunity, MHC class I, Immunoproteasome

## Abstract

Lactic acid bacteria (LAB) possess the ability to argument T cell activity through functional modification of antigen presenting cells (APCs), such as dendritic cells (DCs) and macrophages. Nevertheless, the precise mechanism underlying LAB-induced enhancement of antigen presentation in APCs remains incompletely understood. To address this question, we investigated the detailed mechanism underlying the enhancement of major histocompatibility complex (MHC) class I-restricted antigen presentation in DCs using a probiotic strain known as *Lactococcus lactis* subsp. *Cremoris* C60. We found that Heat-killed-C60 (HK-C60) facilitated the processing and presentation of ovalbumin (OVA) peptide antigen OVA_257-264_ (SIINFEKL) via H-2K^b^ in bone marrow-derived dendritic cells (BMDCs), leading to increased generation of effector CD8+ T cells both *in vitro* and *in vivo*. We also revealed that HK-C60 stimulation augmented the activity of 20S immunoproteasome (20SI) in BMDCs, thereby enhancing the MHC class I-restricted antigen presentation machinery. Furthermore, we assessed the impact of HK-C60 on CD8+ T cell activation in an OVA-expressing B16-F10 murine melanoma model. Oral administration of HK-C60 significantly attenuated tumor growth compared to control treatment. Enhanced Ag processing and presentation machineries in DCs from both Peyer’s Patches (PPs) and lymph nodes (LNs) resulted in an increased tumor antigen specific CD8+ T cells. These findings shed new light on the role of LAB in MHC class-I restricted antigen presentation and activation of CD8+ T cells through functional modification of DCs.

## Introduction

Lactic acid bacteria (LAB) are the predominant commensal bacteria in the small intestine and widely utilized in probiotics, primarily for modulating and stabilizing host immunity (1). Many studies have demonstrated that LAB induce functional modifications in immune cells, primarily focusing on increased cytokine production and enhanced cellular activation in innate immune cells (2). However, the immunomodulatory effects of LAB extend beyond innate immunity, as the functional enhancement of innate immune cells often translate into strengthened adaptive immunity mediated by T cells. Specifically, LAB activation of dendritic cells (DCs) leads to functional modification in these cells, ultimately resulting in enhanced T cell activation (3). The immunological mechanism underlying this T cell modification likely involves LAB signals enhancing the antigen-presenting activity in antigen-presenting cells (APCs), such as DCs and macrophages, characterized by increased cytokine production and upregulation of antigen-presenting molecules and co-stimulatory molecules (4, 5). While, the intricate molecular mechanisms remain unclear.

CD4+ T cells, pivotal in regulating diverse immunological environments including immunological tolerance, anti-inflammatory responses, and acting as a bridge to humoral immunity, are frequently the primary targets of probiotic-mediated T cell functional modification (6). However, it is also documented that LAB can enhance the CD8+ T cells, contributing to beneficial immune responses in cancer immunity (7). CD8+ T cells are primarily cytotoxic lymphocytes (CTLs), crucial in cell-mediated immunity, particularly in combating tumors, viruses and parasites by directly eliminating harmful cells (8). Unlike CD4+ T cell activation, which involves major histocompatibility complex (MHC) Class II molecule-mediated antigen presentation, CD8+ T cell activation is restricted to MHC Class I via APCs (9). MHC Class I-restricted CD8+ T cell activation necessitates an unique antigen processing machinery that utilizes the proteasome system to generate peptide antigens (10). Peptide antigen generation is governed by the 20S immunoproteasome (20SI), possessing distinct structure from the conventional 20S catalytic unit found in 26S proteasomes (11). The 20S proteasome catalytic unit consists of two outer rings containing α-subunits and two inner rings containing β-subunits composed of β1, β2, and β5. In 20SI, these β-subunits are replaced by β1i, β2i, and β5i, respectively. This replacement is induced by pro-inflammatory cytokines, such as interferon-gamma (IFN-γ) and tumor necrosis factor-alpha (TNF-α), activating the proteasome activator (PA) 28, which enhances the catalytic activity of 20SI (12, 13). While this is a well-established mechanism, no studies have addressed whether this machinery could be modulated by exogenous factors such as probiotic LAB.

In this report, we elucidate how probiotic LAB enhances CD8+ T cell-based adaptive immune responses through DC activation. Employing *Lactococcus lactis* subsp. *Cremoris* C60, a probiotic strain known for its ability to activate T cell through APC functional modification (3, 5), we demonstrated how LAB’s stimulatory signals enhance DC antigen presentation activity, consequently promoting CD8+ T cell activation in an antigen-dependent manner. Furthermore, we revealed that orally administrated heat-killed (HK)-C60 enhances CD8+ T cells activity systematically, as evidenced by its suppression of tumor growth in a murine model.

## Materials and methods

### Mice

C57BL/6 mice were purchased from CLEA Japan (Tokyo, Japan) and OT-I transgenic mice (C57BL/6-Tg (TcraTcrb) 1100Mjb/J) were purchased from The Jackson Laboratory (Bar Harbor, ME, USA). All mice were bred in-house and maintained in a specific pathogen-free (SPF) facility with 12-hour light/dark cycles and allowed free access to food and water. To maintain a consistent microbiota and intestinal environment, all mice used in this study were bred in the same facility for at least 3 months. Adult mice of both genders aged 8-12 weeks were used for each experiment. For oral administration, HK-C60 (200 μL of 5.0x10^9^ CFU/mL in saline) or saline (200 μL) was administrated by intragastric using disposable oral gavage needle at every 24 h for 14 days. For establishment of melanoma model, the mice received subcutaneous (s.c.) injection of ovalbumin (OVA)-expressing B16-F10 cells (B16-OVA, 5.0x10^5^ cells) in 100 μl PBS on their back skin. All animal experimental protocols were approved by the animal care and use committee of AIST (protocol No.109), Jichi Medical University (20038-01), University of South China (202005053) and Shibata Gakuen University (2107).

### In vitro antigen processing assay

Bone marrow-derived dendritic cells (BMDCs,1.0x10^6^/mL) were cultured with ovalbumin (OVA) protein (100 μg/mL) or OVA_257-264_ peptide (100 ng/mL). The cultures were further treated with vehicle control (PBS) or HK-C60 (5.0x10^7^ CFU/mL) and incubated at 37°C for 16 h. The expression of SIINFEKL-H-2K^b^ complex recognized by 25-D1.16 monoclonal antibody (mAb) was analyzed by flow cytometry.

### *In vitro* antigen presentation assay

Naive CD8+ T cells were isolated from the spleen of OT-I mice using a Naive CD8+ T Cell Isolation Kit, mouse (Miltenyi Biotec). Naive CD8+ T cells (1.0x10^6^/mL) and BMDCs (1.0x10^5^/mL) were seeded in a 96-well plate with RPMI complete medium in the presence of OVA_252-264_ peptide (100 ng/mL) and recombinant murine interleukin-2 (rmIL-2, 10 ng/mL). The cultures were treated with vehicle control (PBS) or HK-C60 (5.0x10^6^ CFU/mL) and incubated at 37°C for 72 h. The cells were then re-stimulated with PMA (100 ng/mL) and ionomycin (250 ng/mL) in the presence of GolgiStop™ (1 μg/mL) for the last 6 h and subjected to flow cytometry analysis.

### OVA immunization

Mice were orally administered saline (200 μL) or HK-C60 (200 μL of 5.0x10^9^ CFU/mL in saline) for 21 days. On day 7, mice were immunized by subcutaneous (s.c.) injection of OVA protein (100 μg in 100 μL of PBS/50% Complete Freund's Adjuvant (CFA) on their back followed by a boost shot with OVA protein (100 μg in 100 μlL of PBS) by intraperitoneal (i.p.) injection at day 14. On day 21, serum was collected from each mouse and stored at -80°C until use for ELISA. Both spleen and inguinal lymph nodes (LNs) were excised, and single cell suspensions were prepared. The percentages of SIINFEKL-H-2K^b^/Tetramer+ CD8+ T cells were analyzed in the samples by flow cytometry. SIINFEKL-bound H-2K^b^ expression in LNs DCs was also analyzed by flow cytometry. Splenocytes (3.0x10^6^/mL) or LN cells (3.0x10^6^/mL) were restimulated with OVA protein (100 μg/mL) at 37°C for 72 h, the cultured medium was collected and stored at -80°C. IFN-γ concentration in the cultured medium was measured by ELISA.

### In vitro stimulation and antigen treatment for BMDCs

BMDCs (1.0 x 10^6^/mL) were treated with vehicle control (PBS) or HK-C60 (5.0 x 10^7^ CFU/mL) in the presence of OVA protein (100 μg/mL) or OVA_257-264_ peptide (100 ng/mL) at 37°C for 16 h. The expression of cell surface molecules was analyzed by flow cytometry. For antigen uptake assay, BMDCs (1.0 x 10^6^/mL) were treated with vehicle control (PBS) or HK-C60 (5.0 x 10^7^ CFU/mL) in the presence of fluorescein isothiocyanate-conjugated OVA (OVA-FITC) (1 μg/mL) at 37°C for 6 h. The intracellular uptake of OVA-FITC was analyzed by flow cytometry.

### Immunoproteasome activity assay

BMDCs (1.0 x 10^6^/mL) were cultured with OVA protein (100 μg/mL) in the presence of vehicle control (PBS) or HK-C60 (5.0 x 10^7^ CFU/mL) at 37°C for 16 h. Some cultures were treated with ONX-0914 (100 nM) for LMP2 and LMP7 inhibition. After incubation, the cells were washed with PBS and subjected to 20S immunoproteasome (20SI) activity using the 20S Immunoproteasome Activity Assay Kit (ab303734, abcam, Cambridge, United Kingdom). All assay procedures were followed according to the product manual.

### Inhibition of proteasome in antigen processing and presentation assay

The cultures for antigen processing and presentation assays were established by following protocols described in above, then the cultures were further treated with vehicle control (DMSO) or ONX-0914 (100 nM). The treated BMDCs were used for SIINFEKL-H2K^b^ complex expression analysis and Ag-presentation assay against OT-I naïve CD8+ T cells following a protocol represented in above.

## Results

### HK-C60-stimulated BMDCs promote antigen-specific CD8+ T cell generation in MHC class I-restricted manner

**Figure 1:**
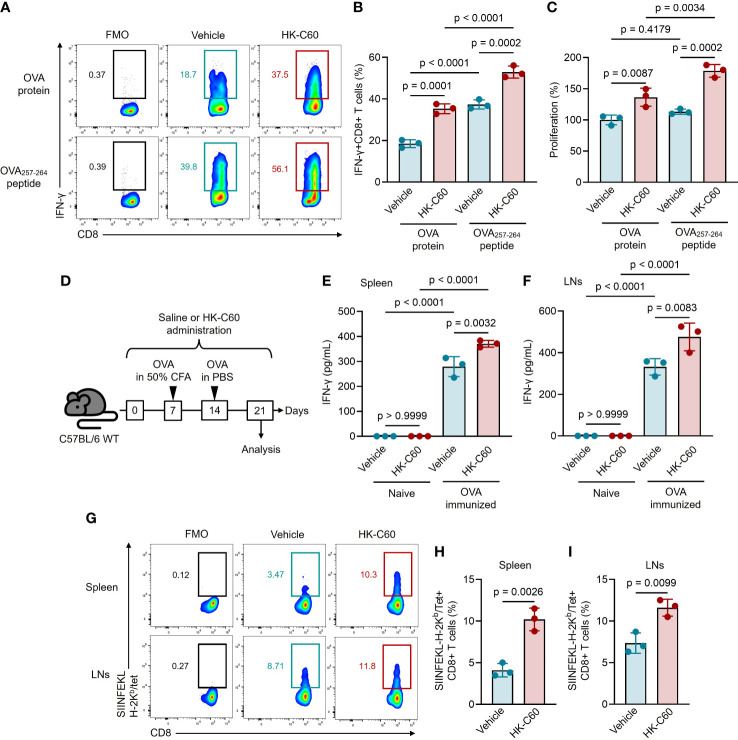
HK-C60-stimulated DCs promote antigen-specific CD8+ T cell activation in an MHC class I-restricted manner in both *in vivo* and *in vitro*. **(A, B)** *In vitro* antigen-presentation assay for CD8+ T cells. OT-I naive CD8+ T cells were co-cultured with BMDCs in the presence of OVA protein or OVA_257-264_ peptide. The cultures were further treated with vehicle (PBS) or HK-C60, and incubated at 37°C for 72 h. The frequencies of antigen specific IFN-γ+CD8+ T cells were analyzed by flow cytometry. Representative plots and cumulative percentages of IFN-γ+CD8+ T cells were shown in **(A, B)**, respectively. **(C)** Proliferation assay for CD8+ T cells. The CD8+ T cell proliferation was measured by MTT assay in the *in vitro* antigen-presentation assay. **(D)** Experimental design for OVA-immunization with or without HK-C60 administration. The mice received oral administration of saline or HK-C60 for 21 days. An immunization was performed with OVA by s.c. injection at day 7 followed by a boost shot with OVA by i.p. injection at day 14. On day 21, the mice were sacrificed and used for analyses. **(E, F)** Antigen restimulation assay. Splenocytes and LN cells isolated from the immunized mice were restimulated with OVA protein at 37°C for 72 h, then IFN-γ concentration in the cultured medium was measured by ELISA. **(G-I)** The frequency of antigen-specific CD8+ T cells. The SIINFEKL-H-2K^b^/Tet+CD8+ T cells in spleen and LNs were ansalyzed in OVA-immunized mice by flow cytometry. Representative plots and cumulative percentages of SIINFEKL-H-2K^b^/Tet+CD8+ T cells were shown in **(G–I)**, respectively. The cumulative data are shown as mean ± SD values of 3 samples. One-way ANOVA or Student’s t-test was used to analyze data for significant differences, and p < 0.05 is considered as significant difference.

To investigate whether HK-C60 stimulation promotes antigen presentation activity in DCs via an MHC class I-restricted manner, we first conducted an *in vitro* antigen presentation assay using BMDCs and naive CD8+ T cells. While we previously reported that HK-C60 promoted the generation of effector CD4+ T cells by enhancing the function of DCs and macrophages (3, 5), we had yet to examine its effect on CD8+ T cell-based adaptive immunity. BMDCs and naive CD8+ T cells isolated from OT-I mice spleens were co-cultured with OVA protein or OVA_257-264_ (SIINFEKL) peptide in the presence or absence of HK-C60. The antigen-presenting ability of DCs was assessed by the generation of IFN-γ+CD8+ T cells. Control cultures generated IFN-γ+CD8+ T cells in the presence of OVA protein or OVA_252-264_ peptide. Addition of HK-C60 significantly increased the percentages of IFN-γ+CD8+ T cells in the cultures (OVA: vehicle 18.5 ± 1.86% vs HK-C60 35.3 ± 2.36%, OVA_257-264_: vehicle 37.3 ± 2.22% vs HK-C60 52.9 ± 2.88%) (**Figures 1A, B**). Cell proliferation was also analyzed, and the results were corresponded to the frequencies of IFN-γ+CD8+ T cells in the cultures, indicating that HK-C60 modified DC function and promoted CD8+ T cell proliferation in an antigen dependent manner (**Figure 1C**). To investigate whether HK-C60 directly stimulates CD8+ T cells, we performed TCR-dependent stimulation with or without HK-C60. Addition of HK-C60 to the culture did not enhance CD8+ T cell activation upon anti-CD3/CD28 mAb stimulation, confirming that the observed stimulatory effect in the presence of HK-C60 is mediated by modified BMDC functions (**Supplementary Figure 1**).

### HK-C60 administration promotes MHC class I-restricted antigen-specific CD8+ T cell generation in a physiological environment

We next investigated whether HK-C60 modulates the activation of CD8+ T cells under physiological conditions. To test this, we orally administered HK-C60 and immunized mice with OVA, then analyzed antigen-specific CD8+ T cell generation. The mice received either oral administration of saline (control) or HK-C60 for 21 days. The primary immunization was performed with OVA protein mixed with 50% of CFA on day 7, followed by a boost shot performed with OVA protein in PBS on day 14. On day 21, the mice were sacrificed and the population of antigen specific CD8+ T cells and their responses were analyzed (**Figure 1D**). Antigen re-stimulation assays showed that IFN-γ production was significantly increased in the splenocyte and LN cell cultures originated from OVA-immunized HK-C60-administered mice as compared to those of OVA-immunized control mice (spleen: saline 279.1 ± 40.0 pg/mL vs HK-C60: 370.5 ± 13.3 pg/mL, LNs: saline 332.1 ± 39.7 pg/mL vs 475.8 ± 66.8 pg/mL) (**Figures 1E, F**). We also investigated antigen-specific CD8+ T cells from OVA-immunized mice, using SIINFEKL-bound H-2K^b^-Tetramer (Tet) (14). The frequencies of SIINFEKL-H-2K^b^/Tet+CD8+ T cells were significantly increased in the spleen and LNs of HK-C60-administered mice compared to those of controls (spleen: saline 4.10 ± 0.81% vs HK-C60 10.2 ± 1.36%, LNs: saline 7.35 ± 1.23% vs 11.6 ± 1.01%) (**Figures 1G-I**).

### HK-C60 Promotes antigen processing and loading on MHC class I molecules in BMDCs

**Figure 2:**
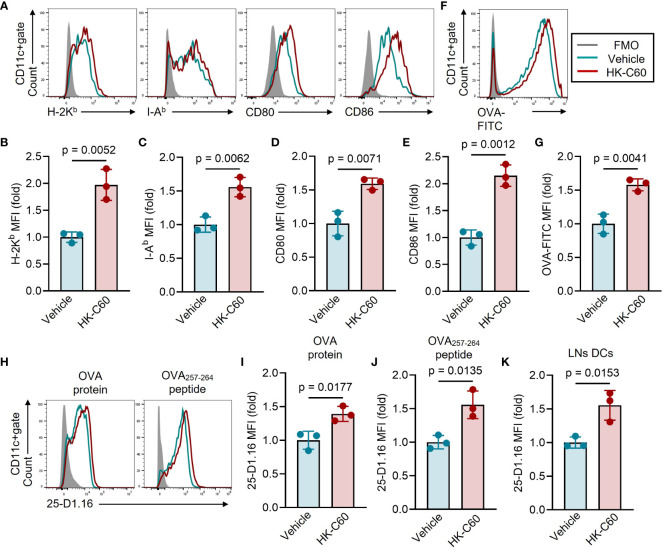
HK-C60 Promotes antigen processing and loading on MHC class I molecules in BMDCs. **(A–E)** Characterization of antigen presenting molecules and co-stimulatory molecule expressions in BMDCs. BMDCs were treated with vehicle (PBS) or HK-C60 in the presence of OVA protein at 37°C for 16 h. The expression of H-2K^b^, I-A^b^, CD80 and CD86 were analyzed by flow cytometry. Representative histogram and cumulative MFI values of target molecules were shown in **(A–E)**, respectively. **(F, G)** Ag -uptaking assay in BMDCs. BMDCs were treated with vehicle (PBS) or HK-C60 in the presence of OVA-FITC at 37°C for 6 h. The intracellularly incorporated OVA-FITC signal was analyzed by flow cytometry. Representative histogram and cumulative MFI values of incorporated OVA-FITC were shown in **(F, G)**, respectively. **(H–K)** Antigen processing efficiency in BMDCs and primary LN DCs. BMDCs were cultured with OVA protein or OVA_257-264_ peptide in the presence of vehicle (PBS) or HK-C60 at 37°C for 16 h. Inguinal LNs cells were isolated from OVA-immunized mice represented in **Figure 1D**. The expression of SIINFEKL-H-2K^b^ complex recognized by 25-D1.16 mAb was analyzed in each sample by flow cytometry. Representative histogram and cumulative MFI values of 25-D1.16 expression were shown in **(H–K)**, respectively. The cumulative data are shown as mean ± SD values of 3 samples. Student’s t-test was used to analyze data for significant differences, and p < 0.05 is considered as significant difference.

Next, we investigated the mechanism by which HK-C60 enhances antigen presenting function of DCs. Firstly, we analyzed the expression of molecules related to antigen processing and presentation in BMDCs upon HK-C60 stimulation. The expression of MHC class I molecule H-2K^b^ was significantly increased in BMDCs in the presence of HK-C60. As we previously reported, the expression of I-A^b^, MHC class II molecule, and CD80 and CD86 co-stimulatory molecules were significantly elevated in HK-C60-stimulated BMDCs compared to controls (**Figures 2A-E**). Next, we assessed the ability of antigen uptake in BMDCs. The BMDCs were incubated with OVA-FITC protein, and the antigen uptake in the presence or absence of HK-C60 was analyzed by flow cytometry. The mean fluorescence intensity (MFI) of OVA-FITC was significantly increased in HK-C60-stimulated BMDCs compared to vehicle-treated cells, indicating that HK-C60 stimulated DCs and promoted OVA protein uptake compared to controls (**Figures 2F, G**). To investigate the efficiency of antigen processing and loading on MHC class I molecules, we analyzed the expression of antigen-bound MHC class I molecules on BMDCs. We used a 25-D1.16 mAb that specifically recognizes the SIINFEKL-H-2K^b^ complex (15). HK-C60 stimulation significantly increased the 25-D1.16 MFI on BMDCs cultured with both OVA protein and OVA_257-264_ peptide compared to control cells, indicating that the expression of the antigen-MHC class I complex was enhanced by HK-C60 stimulation (**Figures 2H-J**). We also investigated the presence of SIINFEKL-H-2K^b^ in DCs in OVA immunized mice. The expression of the antigen-MHC class I complex was significantly increased in the LNs DCs of OVA-immunized mice administered with HK-C60 compared to those administered with saline (**Figure 2K**).

### HK-C60 promotes Ag processing by enhancing immunoproteasome activity

**Figure 3:**
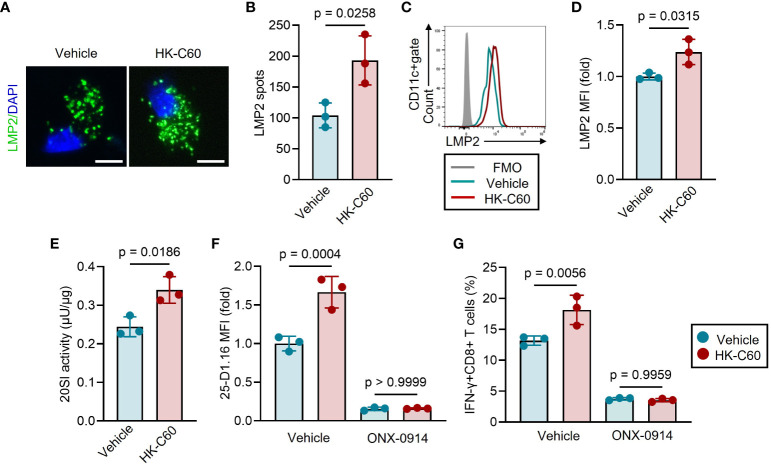
HK-C60 promotes antigen processing by promoting immunoproteasome activity. **(A-D)** 20SI assembly of antigen-fed BMDCs. BMDCs were cultured with OVA protein in the presence of vehicle (PBS) or HK-C60 at 37°C for 16 h. LMP2 was stained in the BMDCs and the number of LPM2 spot was calculated under fluorescence microscope. LMP2 MFI was measured by flow cytometry. Representative IF image and cumulative numbers of LMP2 spots were shown in **(A, B)**, respectively. Bar=10 μm. Representative histogram of LMP2 and cumulative values of MFIs were shown in **(C, D)**, respectively. **(E)** 20SI activity assay in BMDCs. The BMDCs were treated with the same condition represented as experiments of **(A-D)**, and 20SI activity in each sample was measured by using 20S Immunoproteasome Activity Assay Kit. **(F)** Antigen-processing with 20SI inhibition in BMDCs. BMDCs were cultured with OVA protein in the presence of vehicle (PBS) or HK-C60. The culture was further treated with vehicle (DMSO) or ONX-0914, then incubated at 37°C for 16 h. The MFI value of 25-D1.16 was analyzed by flow cytometry. **(G)** Antigen-presentation assay with 20SI inhibition in BMDCs. BMDCs were treated with the same condition represented in **(F)**, then were co-cultured with OT-I naïve CD8+ T cells at 37°C for 16 h. The cumulative data are shown as mean ± SD values of 3 samples. Student’s t-test was used to analyze data for significant differences, and p < 0.05 is considered as significant difference.

To elucidate how HK-C60 stimulation regulates antigen processing in BMDCs, we investigated proteasome assembly and activity in BMDCs, as the regulation of MHC class I-related antigen processing is closely tied to the ubiquitin-proteasome system (9). The BMDCs were incubated with OVA protein, with or without HK-C60, and the assembly of 20S immunoproteasome (20SI) was examined by using immunofluorescence and flow cytometry. We stained LMP2, a β-subunit of 20SI with chymotrypsin activity (16), and the number of LMP2 spots was counted in fluorescence microscopic images to assess 20SI assembly in BMDCs. HK-C60 stimulation significantly increased the number of LMP2 spots compared to vehicle treatment in OVA-fed BMDCs (**Figures 3A, B**). The expression level of LMP2 was further quantified by flow cytometry analysis revealed that HK-C60 stimulation significantly elevated the expression of LMP2 compared to vehicle treatment in BMDCs (**Figures 3C, D**).

Furthermore, we measured 20SI activity in both HK-C60 and vehicle-treated BMDCs. BMDCs stimulated with HK-C60 exhibited higher 20SI activity than vehicle-treated cells (**Figures 3E**; **Supplementary Figure 2**). To ascertain the importance of 20SI in C60-mediated enhanced antigen presentation, we inhibited its activity using a specific inhibitor, ONX-0914 (17). The inhibition of 20SI significantly downregulated the expression of the SIINFEKL-H-2K^b^ complex, as detected by the 25-D1.16 mAb, in OVA-fed BMDCs. This 20SI inhibition abolished the upregulation of the antigen-MHC class I complex expression in HK-C60-stimulated BMDCs resulting in comparable 25-D1.16 MFI values to those of control samples (**Figure 3F**). Moreover, 20SI inhibition also downregulated the antigen-specific activation of CD8+ T cells in HK-C60-stimulated BMDCs. HK-C60-stimualted BMDCs treated with ONX-0914 generated a low frequency of IFN-γ+CD8+ T cells in the antigen presentation assay that was equivalent to vehicle control treated BMDCs. (**Figure 3G**).

### HK-C60 administration systemically activates DC function and promotes subsequent CD8+ T cell activation by enhancing anti-tumor response in a murine melanoma model

**Figure 4:**
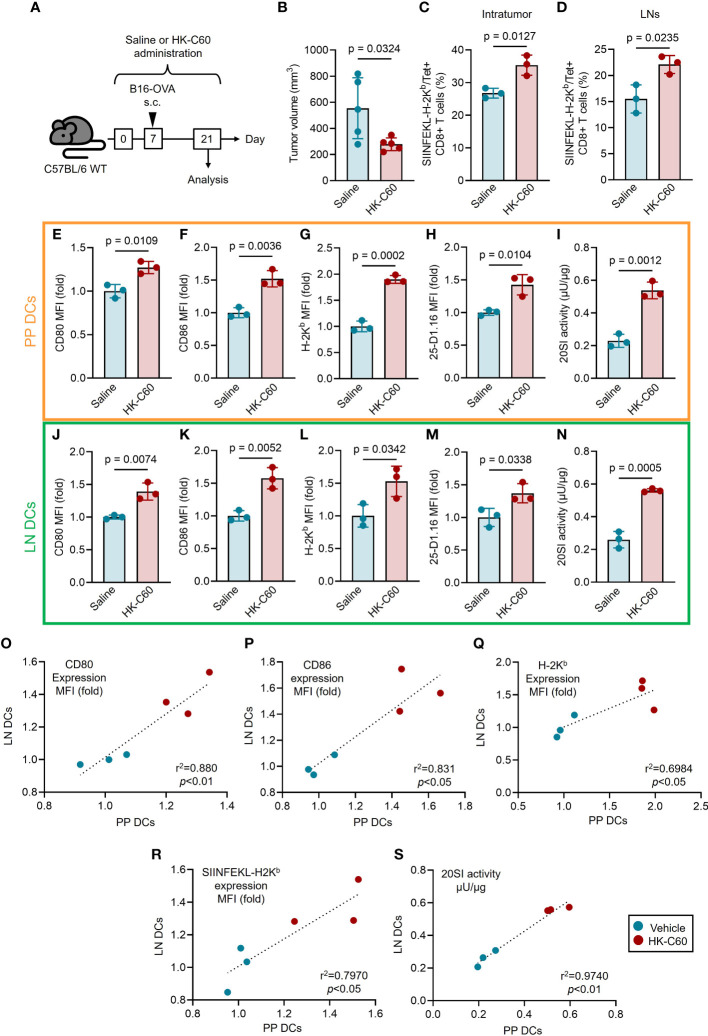
HK-C60 administration suppresses tumor growth through functional activation of DCs not only in the intestine but also in systemic manner. **(A)** Experimental design of murine melanoma model. The mice received oral administration of saline or HK-C60 for 21 days. B16-OVA cells were inoculated by s.c. injection in day 7. The mice were sacrificed after 14 days (21 days of saline or HK-C60 administration) and used for analysis. **(B)** The volumes of tumors (day 14). **(C, D)** The percentages of tumor antigen specific CD8+ T cells in tumor bearing mice. Tumor and iLNs were excised, then the isolated cells were used for flow cytometry analyses. The percentages of TRP-2/Tet+CD8+ T cells in intratumor (IT) and iLNs were represented in **(C, D)**, respectively. **(E-G, J-L)** DCs activities in tumor bearing mice. CD80, CD86 and H-2K^b^ expressions in PP DCs **(E-G)** and iLN DCs **(J-L)**. 25-D1.16 expressions in PP DCs **(H)** and iLN DCs **(M)**. 20SI activities in PP DCs **(I)** and iLN DCs **(N)**. Correlation of DC activities between PP DCs and iLN DCs of tumor bearing mice **(O-S)**. The cumulative data are shown as mean ± SD values of 3 or 5 samples. Student’s t-test was used to analyze data for significant differences, and *p* < 0.05 is considered as significant difference.

Finally, we investigated the contribution of the HK-C60-mediated strengthen DC function in CD8+ T cell-based immunity under physiological environment using a murine melanoma model. The mice received oral administration of HK-C60 or saline for 21 days, and B16-OVA cells were inoculated into the mice on day 7. After 14 days of tumor inoculation (day 21), the mice were sacrificed and used for analyses (**Figure 4A**). Tumor growth was significantly suppressed in HK-C60 administrated mice compared with saline administrated mice (Saline: 554.5 ± 223.6 mm^3^ vs HK-C60: 278.6 ± 50.6 mm^3^) (**Figure 4B**). The frequencies of tumor antigen specific CD8+ T cells, detected as SIINFEKL-H-2K^b^/Tet+ cells, were significantly increased in both tumor microenvironment (TME) and inguinal LNs of HK-C60 administrated mice compared to control mice (**Figures 4C, D**). To investigate whether the immune modification by HK-C60 was distributed systemically from the gut, we compared DC activities between PP and inguinal LN in the tumor baring mice. Consistent with the results obtained from *in vitro* experiments using BMDCs represented in **Figures 3**, **4**, the expressions of antigen presentation related molecules, such as CD80, CD86 and H-2K^b^, as well as SIINFEKL-H-2K^b^ complex were all significantly increased in PP DCs by HK-C60 administration as compared to those of saline administration. Additionally, 20SI activity was also increased in PP DCs of HK-C60 administrated mice (**Figures 4E-I**). The functional modification of intestinal DCs was subsequently reflected into the activity of CD8+ T cells, meaning that IFN-γ or TNF-α producing populations were increased in CD8+ T cells of PPs by HK-C60 administration (**Supplementary Figures 3A, B**). These functional parameters investigated in PP DCs were all significantly increased in LN DCs of HK-C60 administrated mice compared with saline administrated mice (**Figures 4J-N**). Interestingly, there were strong positive correlations in CD80, CD86, H-2K^b^, SIINFEKL-H-2K^b^ complex expressions and 20SI activity between PP DCs and inguinal LN DCs, implying that HK-C60 oral administration induces functional modification on DCs not only in the intestinal environment but also in systemically (**Figures 4O-S**).

## Discussion

Many studies, including our previous work, have elucidated the mechanism of T cell activation modulated by probiotics focusing on the enhanced immune function and antigen-presenting activity of APCs (3, 5). Therefore, the characterization of cytokine production and expression levels of antigen presentation-related molecules have been considered indispensable parameters for evaluating APC activity. While these functional upregulations may impact the antigen-presenting activity of APCs and consequently enhance T cell function, they do not fully explain how LAB’s stimulatory signals work for antigen processing and presentation on APCs. In this study, we provide the first evidence explaining a part of the molecular mechanism behind LAB-mediated enhanced antigen presentation by APCs. Although our results are limited to MHC class I-restricted antigen presentation to CD8+ T cells, this study provides strong evidence that LAB are able to enhance antigen presentation by modification of antigen processing machinery via immunoproteasome in APCs. While We have not shown the triggering factor for 20SI activation in HK-C60 exposed DCs. Previous studies reported that inflammatory cytokines, such as TNF-α and IFN-γ, drive 20SI activation (12, 13). In fact, our previous report showed that TNF-α productions were increased in DCs and macrophages by HK-C60 stimulation, while we did not show the participation of IFN-γ (3, 5). We have not yet investigated the intracellular signaling pathway and the detailed mechanism of the conversion of the 20S subunit. For instance, investigating the activation of signal transducer and activator of transcription 1 (STAT1) and nuclear factor kappa-light-chain-enhancer of activated B cells (NF-κB), which are downstreams of these cytokine signals, as well as characterizing downstream gene expressions, would provide a more solid understanding (18, 19). Additionally, the investigation of the proteasome activator (PA) 28 is also important. Previous reports have shown that pro-inflammatory cytokines activate PA28, and this process is indispensable for subunit conversion and the formation of 20SI. Moreover, PA28 is a crucial factor for 20SI activation and protein degradation, as it can form a PA28-20SI or PA28-20SI-19S complex (20, 21). In particular, the PA28-20SI-19S hybrid proteasome is considered a specific proteasome for MHC class I peptide antigen processing (21).

From a translational perspective, our study presents a suppressive effect of HK-C60 administration against tumor growth in a murine B16-OVA derived melanoma (**Figures 4A, B**). This effect is based on the functional enhancement of DCs in both the intestinal and systemic compartments in HK-C60 administrated tumor bearing mice (**Figures 4E-N**). Moreover, this modified DC function surely transferred to activation and increase of tumor antigen specific CD8+ T cells (SIINFEKL-H-2K^b^/Tet+CD8+ T cells), which are primary killing players in the anti-tumor immunity (**Figures 4C, D**). The data strongly supports the evidence that the probiotic LAB-originated immuno-modification in the intestinal environment is distributed to whole body and regulates the anti-tumor response based on CD8+ T cells. Although we found the positive correlation of the functional activation status between PP DCs and LNs DCs in HK-C60 administrated tumor bearing mice, we have not explained yet how the experience of HK-C60-based immunological modification in the gut is transferred to peripheral immunity which is eventually fighting with tumor at the front line (**Figures 4O, S**). We hypothesized that the information of immunologically modified intestinal environment is transferred to bone marrow and/or peripheral via other immune cells, such and neutrophils and monocytes both of which can travel by blood stream. In fact, recent studies reported that these myeloid cells are able to regulate other immune cells functions in diverse responses including anti-tumor immunity (22, 23). Especially, monocytes can be frequently differentiated to DCs (monocyte-derived DCs; moDCs) and are involved in T cells activation (23). We also consider about the change of microbiome by HK-C60 administration which influences to systemic immunity. In fact, it has gradually been recognized that altered microbiome in probiotics modify anti-tumor immunity (24). Further investigation must be required to reveal how modified intestinal information is transferred and regulate peripheral DC function in HK-C60 administrated mouse. Interestingly, our results align with recent report demonstrating that *Lactobacillus reuteri* can modulate immune responses and enhance the efficacy of immune checkpoint blockade therapy (7). While the mechanisms underlying these effects may differ from our present study, the evidence hints at the potential ability of various LAB strains to suppress cancer through diverse immune modifications. Moreover, our findings open the possibilities beyond the scope of cancer research. For instance, it is well-established that aging triggers proteasome dysfunction and the accumulation of cellular debris in the cytosol (25). As proteasome dysfunction underlies specific diseases (26–28), the activation of proteasomes using LAB could offer a novel approach to address complex disease symptoms.

In conclusion, our study provides novel insight into probiotic LAB and functional modification of DCs. This finding could explain a detailed mechanism of CD8+ T cell activation by enhanced MHC class I-restricted antigen presenting machinery in LAB stimulated DC. Although further research is still needed to figure out the best usage of probiotic LAB in targeting functional modification of DCs, our finding has a strong potential which can be adopt to translational use not only for cancer, but also some diseases.

## Data availability statement

The original contributions presented in the study are included in the article/**Supplementary Material**. Further inquiries can be directed to the corresponding authors.

## Ethics statement

The animal study was approved by All animal experimental protocols AIST (protocol No.109) were approved by the animal care and use committee of Jichi Medical University (20038-01), University of South China (202005053) and Shibata Gakuen University (2107). The study was conducted in accordance with the local legislation and institutional requirements.

## Author contributions

SS: Writing – review & editing, Writing – original draft, Visualization, Validation, Supervision, Resources, Project administration, Methodology, Investigation, Funding acquisition, Formal analysis, Data curation, Conceptualization. ZP: Writing – review & editing, Resources, Methodology, Investigation, Formal analysis, Data curation. DYC: Writing – review & editing, Visualization, Formal analysis, Data curation. AO: Writing – review & editing, Writing – original draft, Visualization, Validation, Supervision, Resources, Project administration, Investigation, Funding acquisition, Formal analysis, Data curation, Conceptualization. NMT: Writing – review & editing, Supervision, Resources, Conceptualization, Funding acquisition.
