# Supplementary material for: Probiotic lactic acid bacteria promote anti-tumor immunity through enhanced major histocompatibility complex class I-restricted antigen presentation machinery in dendritic cells
Source: Front Immunol. 2024 Mar 27;15:1335975. doi: 10.3389/fimmu.2024.1335975 (PMC11008462; doi:10.3389/fimmu.2024.1335975)
Supplement: Supplementary file 1 [file DataSheet_1.docx]

**Supplemental information**

**Supplemental Figures**

**
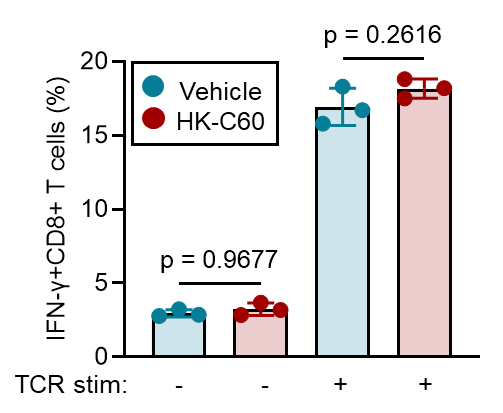
**

**Supplemental Figure 1. TCR stimulation for CD8+ T cells in the presence of HK-C60**

OT-I naive CD8+ T cells (1.0x10^6^/mL) were cultured with or without TCR stimulation (1.0x10^6^/mL of anti-CD3/CD28 microbeads) in the presence or vehicle (PBS) or HK-C60 (5.0x10^7^ CFU/mL) at 37℃ for 72 h. The frequency of IFN-γ+CD8+ T cells was analyzed by flow cytometry. The cumulative data is shown as mean ± SD values of 3 samples. Student’s t-test was used to analyze data for significant differences, and *p* < 0.05 is considered as significant difference.

**
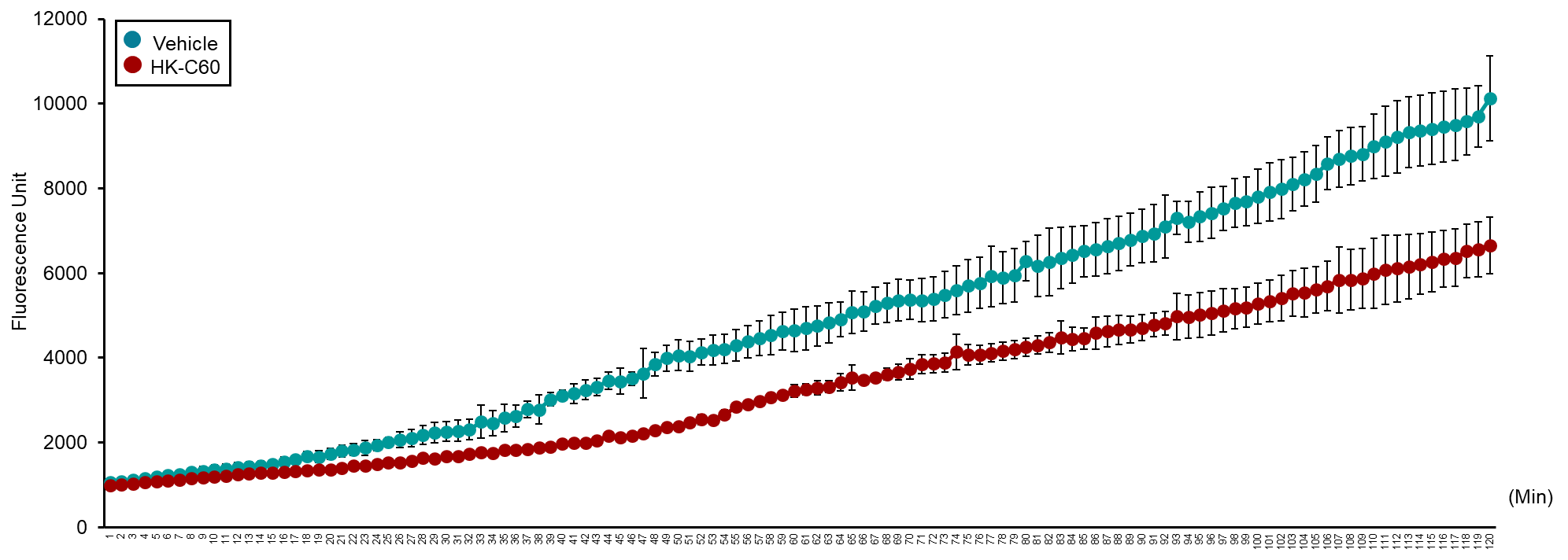
**

**Supplemental Figure 2. 20SI activity assay in antigen-fed BMDCs**

BMDCs (1.0 x 10^6^/mL) were cultured with OVA protein (100 μg/mL) in the presence of vehicle control (PBS) or HK-C60 (5.0 x 10^7^ CFU/mL) at 37°C for 16 h. 20SI activity was measured by using the 20S Immunoproteasome Activity Assay Kit. The RFU plot of each assay time point is shown as mean ± SD values of 3 samples.


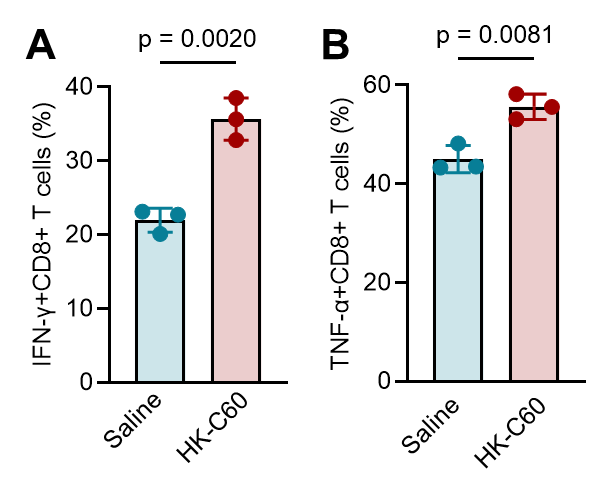


**Supplemental Figure 3. Cytokine production in PP CD8+ T cells**

PP cells were isolated from tumor bearing mice received saline or HK-C60 oral administration for 21 days. The CD8+ T cells were stimulated with anti-CD3 mAb and anti-CD28 mAb for 72 h, then IFN- γ and TNF-α productions were analyzed by flow cytometry. The cumulative data is shown as mean ± SD values of 3 samples. Student’s t-test was used to analyze data for significant differences, and *p* < 0.05 is considered as significant difference.

**Supplemental Protocols**

*Reagents and antibodies*

Phorbol 12-myristate 13-acetate (PMA), ionomycin and ONX-0914 were purchased from Sigma Aldrich (St. Louis, MO, USA). GolgiStop^TM^ was purchased from BD bioscience (Franklin lakes, NJ, USA). Recombinant murine granulocyte-macrophage colony-stimulating factor (rmGM-CSF) was purchased from Peprotech (Cranbury, NJ, USA). Ovalbumin (OVA) and OVA_257-264_ peptide, anti-CD3/CD28 microbeads were purchased from Thermo Fisher Scientific (Waltham, MA, USA). Anti-CD11c (N418), anti-CD80 (16-10A1), anti-CD86 (GL-1), anti-I-A^b^ (AF6-120.1), anti-H-2K^b^ (AF-6-88.5), anti-CD3 (17A2), anti-CD4 (GK1.5), anti-CD8a (53-6.7), anti-IFN-γ (XMG1.2), anti-TNF-α (MP6-XT22), anti-H-2K^b^ bound SIINFEKL (25-D1.16) and anti-CD16/CD32 (2.4G2) were purchased from BioLegend (San Diego, CA, USA). SIINFEKL-H-2K^b^/Tetramer was purchased from MBL (Tokyo, Japan). Anti-LMP2 (ab184172) and goat anti-rabbit IgG H&L (ab150077)-Alexa 488 were purchased from Abcam (Cambridge, UK).

*Lactic Acid Bacteria Culture*

*Lactococcus lactis* subsp. *Cremoris* C60 was cultured in MRS broth (BD Difco^TM^, BD Bioscience, Franklin Lakes, NJ, USA) at 30 ℃ for 24 h. The bacterial colony forming unit (CFU/mL) was calculated in each culture. For HK-C60 preparation, the bacteria were autoclaved at 95 ℃ for 10 min, then the bacterial cells were precipitated by centrifugation at 5,000 *g* for 10 min. After being washed with saline (0.9% NaCl), the pellet was finally suspended in saline (for in vivo administration) or PBS (for in vitro). The suspension was used as HK-C60 for each experiment.

*Cell isolation*

BM cells were flushed from femur and tibia in RPMI complete medium (RPMI 1640 supplemented with 10% fetal bovine serum (FBS), 100 U/mL penicillin, and 100 U/mL streptomycin) by using 10 mL syringe with 27G needle. The cells were washed, and contaminated red blood cells (RBCs) were eliminated by treating with RBC lysis buffer at RT for 10 min. After washed the cells with RPMI complete medium, the cells were collected by centrifugation at 300 *g* for 5 min and the cells were used as BM-isolated cells. Splenocytes were prepared from spleen by mechanical crush on a 70 μm of cell strainer in RPMI complete medium. The cells were washed and contaminated RBCs were eliminated by treating with RBC lysis buffer at RT for 10 min, the cells were collected by centrifugation at 300 *g* for 5 min and the cells were used as splenocytes. Naive CD8+ T cells were isolated by using naive CD8+ T cell isolation kit (Miltenyi Biotach, Bergisch Gladbach, North Rhine-Westphalia, Germanya). All procedures were followed by product manual. The samples with more than 90 % of CD62L+CD44- cells in CD8+ T cell population in flow cytometric evaluation were used for subsequent experiments. Lymph node (LN) cells were isolated from inguinal LN (iLN) by mechanical crush on a 70 μm of cell strainer in RPMI complete medium. The cells were washed and collected by centrifugation at 300 *g* for 5 min and the cells were used as LN-isolated cells. Peyer's patch (PP) cells were isolated from small intestinal PPs. First, the excised PPs were treated with epithelial dissociation buffer (PBS containing 10% FBS, 100 mg/mL penicillin, 100 mg/mL streptomycin, 20 mM EDTA and 10 mM dithiothreitol (DTT)) at 37℃ for 20 min with staring followed by treating with digestion buffer (RPMI complete medium containing 1 mg/mL collagenase type-I, 50 μg/mL DNase) and incubated at 37 °C for 30 min with staring. After digestion, the PPs were collected by decantation onto a 70 μm cell strainer, then mechanically crushed by mechanically in RPMI complete medium. Finally, the cells were washed with RPMI complete medium and filtered through a 40 μm cell strainer followed by centrifugation at 300 *g* for 5 min. The collected cells were used as PP isolated cells. For analysis of tumor infiltrated CD8+ T cells, an excised tumor was chopped and mechanically crushed on a 70 μm cell strainer, then washed once with RPMI complete medium. The samples were treated with 1 mg/mL of collagenase I at 37℃ for 30 min. After digestion, the cells were washed with RPMI complete medium and collected by centrifugation at 300 *g* for 5 min. The collected tumor-isolated cells were used for analysis.

*Bone marrow dendritic cells (BMDCs) preparation*

BM-isolated cells were isolated from the tibia and femur of C57BL/6 mice (8-10 weeks) and seeded at 3.0x10^5^/mL in RPMI complete medium containing rmGM-CSF (20 ng/mL). The cells were cultured at 37°C for 8 days with medium replaced half-volume on days 3 and 6. On day 8, floating cells were harvested as BMDCs and washed with RPMI complete medium. The quality of BMDC differentiation was assessed by flow cytometry, and samples with more than 90% of CD11c+ cells were used for subsequent experiments.

*Immunofluorescence*

BMDCs (2.5x10^6^/mL) were seeded in chamber slide with OVA protein (100μg/mL) and treated with vehicle control (PBS) or HK-C60 (5.0x10^7^ CFU/mL) at 37℃ for 16 h. The cells were fixed with 4% paraformaldehyde (PFA) at RT for 10 min, then were washed with PBS followed by blocking with 5% bovine serum antigen (BSA)/PBS at RT for 30 min. LMP2 staining was performed by mouse anti-LMP2 antibody (1:100) at 4℃ overnight followed by secondary Ab treatment anti-rabbit IgG H&L-Alexa 488 (1:2500) at RT for 30 min. The slide was sealed with ProLong™ Gold Antifade Mountant (Thermo Fischer Scientific) and observed by fluorescence microscope (BZ-X700, KEYENCE, Tokyo, Japan).

*Flow cytometry*

Flow cytometry analyses were performed by using flow cytometers (LSR Fortessa SORP and FACS Aria I (BD Biosciences). For the surface markers staining, the cells were first incubated with FcR blocker (anti-CD16/CD32) at 4℃ for 10 min followed by incubation with the fluorochrome-conjugated antibodies in PBS/2 % FBS at 4℃ for 30 min. Intracellular staining was performed by using BD Cytofix/Cytoperm kit by following manufacture’s instruction. Briefly, extracellular marker-stained samples were fixed at 4℃ for 20 min, then intracellular protein was stained with antibodies at 4℃ for 30 min. Obtained data were analyzed by FACS Diva (BD Bioscience) and FlowJo (BD Bioscience).

*Enzyme-linked immunosorbent assay (ELISA)*

The cytokine concentration in the cultured medium was measured by ELISA by using DuoSet ELISA kit (R&D Systems, Minneapolis, MN, USA). All procedures were performed by following manufacture’s instruction.

*Statistics*

Statistical analyses were performed by using GraphPad Prism 9.0 (GraphPad Software, San Diego, CA, USA). Student t-test and one-way analysis of variance (ANOVA) were used to analyze the data for significant differences.
